# Course of frailty stratified by physical and mental multimorbidity patterns: a 5-year follow-up of 92,640 participants of the LifeLines cohort study

**DOI:** 10.1186/s12916-021-01904-x

**Published:** 2021-02-08

**Authors:** R. C. Oude Voshaar, H. W. Jeuring, M. K. Borges, R. H. S. van den Brink, R. M. Marijnissen, E. O. Hoogendijk, B. van Munster, I. Aprahamian

**Affiliations:** 1 Department of Psychiatry, University of Groningen, University Medical Center Groningen, Groningen, The Netherlands; 2 Department and Institute of Psychiatry, University of São Paulo (USP), São Paulo, Brazil; 3 Department of Epidemiology and Data Science, Amsterdam Public Health research institute, Amsterdam UMC - location VU University Medical Center, Amsterdam, The Netherlands; 4 Department of Internal Medicine, University of Groningen, University Medical Center Groningen, Groningen, The Netherlands; 5 Geriatrics division, Internal Medicine Department, Faculty of Medicine of Jundiaí, Group of Investigation on Multimorbidity and Mental Health in Aging (GIMMA), Jundiaí, Brazil

**Keywords:** Multimorbidity, Frailty, Frailty index, Mortality, Biological aging

## Abstract

**Background:**

The frailty index (FI) is a well-recognized measurement for risk stratification in older people. Among middle-aged and older people, we examined the prospective association between the FI and mortality as well as its course over time in relation to multimorbidity and specific disease clusters.

**Methods:**

A frailty index (FI) was constructed based on either 64 (baseline only) or 35 health deficits (baseline and follow-up) among people aged ≥ 40 years who participated in LifeLines, a prospective population-based cohort living in the Northern Netherlands. Among 92,640 participants, multivariable Cox proportional hazard models were fitted to study the hazard ratio (HR) of the FI at baseline, as well as for 10 chronic disease clusters for all-cause mortality over a 10-year follow-up. Among 55,426 participants, linear regression analyses were applied to study the impact of multimorbidity and of specific chronic disease clusters (independent variables) on the change of frailty over a 5-year follow-up, adjusted for demographic and lifestyle characteristics.

**Results:**

The FI predicted mortality independent of multimorbidity and specific disease clusters, with the highest impact in people with either endocrine, lung, or heart diseases. Adjusted for demographic and lifestyle characteristics, all chronic disease clusters remained independently associated with an accelerated increase of frailty over time.

**Conclusions:**

Frailty may be seen as a final common pathway for premature death due to chronic diseases. Our results suggest that initiating frailty prevention at middle age, when the first chronic diseases emerge, might be relevant from a public health perspective.

**Supplementary Information:**

The online version contains supplementary material available at 10.1186/s12916-021-01904-x.

## Background

Public health policies need to better address gerontological particularities to deliver patient-centered care. By the age of 65, more than half of the population already has multiple chronic diseases, which increases to over 80% in those aged 80+ [1]. As an evaluation of the concept of comorbidity, referring to an index disease in the presence of other diseases, multimorbidity is defined as the presence of two or more chronic diseases without assigning one of the diseases as the index disorder. Multimorbidity has major consequences including functional impairment, poor quality of life, and high health care utilization and costs [2, 3] and challenges traditional health care systems based on a single-disease-oriented approach [4]. Unfortunately, randomized controlled trials that test the effectiveness of health-service or patient-oriented interventions to improve outcomes in people with multimorbidity are still inconclusive [5, 6]. This may be explained by the inclusion of a heterogeneous population in terms of number, type, and severity of underlying diseases and argues for risk stratification to identify which patients might benefit from a given intervention [4, 7]. Frailty is increasingly recognized as an important concept for risk stratification to prevent further decline and iatrogenic harm [8]. Frailty is characterized by functional decline in multiple physiological systems leading to poor resolution of homeostasis after a stressor [8]. Epidemiological studies have consistently demonstrated that frailty is an independent risk factor for adverse health events, such as falls, disability, hospitalization, admission to long-term care facilities, and mortality [8].

Frailty may be a good candidate for risk stratification in the case of multimorbidity. Meta-analyses have shown that the prevalence of multimorbidity in frail people is 72%, while only 16% of people with multimorbidity are frail [9]. One of the most common operationalizations of frailty for research purposes is based on the deficit accumulation model. This model postulates that the proportion of aging-related health deficits, i.e., the frailty index (FI), reflects biological age over chronological age [10]. An FI can be reliably constructed in ongoing epidemiological studies provided that it includes at least 30 health deficits (signs, symptoms, diseases, disabilities, abnormal test results) that are associated with health status, prevalent in at least 1% of the sample, increase in prevalence with age but do not saturate too early (before 65 years), and cover a range of systems when considered as a group [11]. Of importance, the characteristics of the FI are independent of the specific health-deficits included. In other words, the FI may still be relevant for patients with age-related chronic diseases which are included as one of the many health deficits in an FI. It is assumed that older people accumulate deficits at a rate of 3% per year on average [11, 12]. The relevance and impact of frailty, however, may not be limited to older people, since chronic somatic diseases often emerge in midlife and frailty may be more easily reversed in its earlier stages [12]. However, large prospective cohort studies on frailty have rarely included middle-aged people [13, 14]. Moreover, increases in frailty over time, stratified by chronic disease status or multimorbidity, have never been examined. This knowledge is of interest from a public health perspective as well as for developing care pathways [15].

There are four objectives of this paper: (1) to identify frailty, using the FI, in a large cohort of people aged 40 years and over; (2) to examine the relationship of frailty, measured using the FI, with 10-year mortality rates; (3) to examine whether the association between the FI and mortality is independent of multimorbidity (number of diseases as well as specific physical and mental health disease clusters) and/or interacts with multimorbidity; and (4) to examine whether multimorbidity or specific disease clusters are associated with an accelerated increase of frailty at a 5-year follow-up. We primarily hypothesize that frailty is a stronger and age-independent predictor of mortality than multimorbidity or any disease cluster. Secondly, we hypothesize that most chronic disease clusters are associated with an increase of frailty over time.

## Methods

### Lifelines

LifeLines is a multi-disciplinary prospective population-based cohort study that uses a unique three-generation design to examine the health and health-related behaviors of 167,729 people living in the northern Netherlands. The baseline assessment took place between 2006 and 2013 and LifeLines aims to track these people over a 30-year period with site-visits every 5 years. It employs a broad range of investigative procedures to assess the biomedical, behavioral, physical, and psychological factors that contribute to the health and disease of the general population, with a special focus on multimorbidity and complex genetics [16].

The LifeLines Cohort Study is being conducted according to the principles of the Declaration of Helsinki and in accordance with research code of the University Medical Center Groningen (UMCG). All participants have signed informed consent and the LifeLines study has been approved by the medical ethical committee of the UMCG, The Netherlands.

### Recruitment

General practitioners (GPs) invited patients between the ages of 25 and 50 years except those with limited life expectancy (< 5 years) and/or insufficient knowledge of the Dutch language. Participants received a baseline questionnaire and visited one of the LifeLines research sites for a physical examination, including lung function, ECG, and cognitive tests, and completed extensive questionnaires. Within 1 week, a fasting blood sample was collected. Linkage with medical registries and environmental data is being established.

During the baseline visit, family members of participants were also invited to participate in LifeLines. In addition, inhabitants of the northern provinces could also register via the LifeLines website. In total, 49% (81,652/167,729) of the included participants were invited through their GP, 38% (64,489/167,729) were recruited via participating family members and 13% (21,588/167,729) self-registered via the LifeLines website. The LifeLines adult study population appears to be broadly representative of the adult population of the Northern Netherlands [17].

For the present study, we selected participants aged 40 years and over (*n* = 96,127) a priori. Of these pre-selected people, a total of 3487 (3.6%) had missing data with respect to multimorbidity and/or frailty, which resulted in a final sample size of 92,640 people.

### Construction of the frailty index (FI)

Following the guidelines of Searle and colleagues [11], health deficits were included in the LifeLines–FI if they (a) were biologically meaningful in representing multiple organ systems, and (b) accumulated with age, but were not overly prevalent at some younger age, and (c) had less than 5% missing values. Based on the LifeLines data catalog, chronic somatic diseases (17 items), physical measurements (9 items), disability (10 items), subjective health measurements (12 items), sensory function (2 items), mental health indicators (4 items), neuropsychological markers (6 items), and blood biomarkers (22 items) were considered for inclusion in the LifeLines-FI. Each deficit was coded as 0 (absence) or 1 (presence) or when clinically relevant, as any number between 0 and 1. For clinical interpretation, people can be classified as robust (FI < 0.08), pre-frail (0.08 ≤ FI < 0.25), and frail (FI ≥ 0.25) [18].

This procedure resulted in a baseline LifeLines-FI consisting of 64 items (FI-64). Since some key variables are not available at the 5-year follow-up, we also constructed a 35-item LifeLines-FI (FI-35) using similar health deficits at baseline and follow-up, thereby allowing longitudinal monitoring of frailty severity [11]. Additional file 1 provides an overview of the health deficits included in the FI-64 and FI-35.

### Disease domains

Chronic diseases were based on a self-report questionnaire and were clustered as follows: (1) psychiatric disorders (chronic fatigue syndrome, depression, burn-out, social phobia, agoraphobia, panic disorder, anxiety disorder, bipolar disorder, schizophrenia, eating disorder, obsessive-compulsive disorder, ADHD), (2) lung diseases (asthma, chronic obstructive pulmonary disease), (3) heart disease (myocardial infarction, established coronary artery disease, arrhythmias, heart failure, aneurysm), (4) brain disorders (migraine, epilepsy, multiple sclerosis, spasticity, Parkinson’s disease, dementia), (5) cerebrovascular disease (stroke, clinically relevant carotid stenosis), (6) gastrointestinal diseases (Crohn’s disease, ulcerative colitis, hepatitis, liver cirrhosis), (7) kidney diseases, (8) endocrine diseases (thyroid disease, diabetes mellitus), (9) musculoskeletal disease (osteoarthritis, rheumatoid arthritis, osteoporosis), and (10) cancer (any type). A multimorbidity score was calculated based on the sum of positive disease clusters (range 0–10).

### All-cause mortality

Year of death up to 2019 was retrieved by linkage with the national mortality data as collected in the database of Statistics Netherlands (see cbs.nl). This enabled us to calculate yearly survival rates by subtracting the year of death by the year of the baseline assessment.

### Covariates

We included age, gender, and level of education (elementary, middle and higher) as socio-demographic characteristics. We included partner status (yes/no), body mass index (kg/m^2^), use of alcohol, and smoking as lifestyle characteristics which might independently contribute to mortality rates and frailty trajectories. Use of alcohol was classified as drinking no alcohol at all, using moderate/social levels of alcohol, or using problematic levels of alcohol (≥ 21 drinks a week). Smoking was classified as never, ever, or current.

### Analyses

We examined the distribution of the FI-64 as well as its associations with age, gender, and the baseline FI-35. Multivariable Cox proportional hazard models were fitted to study the association between the FI-64 score (as well as all chronic disease clusters in separate models) and all-cause mortality. Hazard ratios (HR) and 95% confidence intervals (95% CI) were reported for the total population. Survivors were censored at 10-year follow-up. Subsequently, the interaction between the FI-64 and each chronic disease cluster was tested to explore whether the impact of the FI is conditional on the disease cluster in question. Interactions of age and gender with the FI and all chronic disease clusters were explored, and in cases of significance, stratified results were present by either age (40–59 versus ≥ 60 years) or gender.

Lastly, the impact of baseline multimorbidity or specific somatic disease clusters (independent variables) was examined in regard to their impact on the change of frailty over time using linear regression analyses. The FI-35 at follow-up was included as the dependent variable, adjusted for the baseline FI-35 and covariates.

All multivariate analyses included either the demographic variables age, gender, and level of education (model 1) or age, gender, level of education, and all lifestyle characteristics (model 2). The number of people with missing values was low and varied between 0 and 438 (0.47%) (see footnote of Table 1 for the exact numbers per variable). Therefore, multivariate analyses were conducted listwise, excluding participants with missing data on any of the covariates.

**Table 1:** Basic characteristics of study sample (*n* = 92,640)

Characteristics:		
*Demographics:*		
• Age (years)	mean (SD)	51.4 (8.9)
• Female gender	*n* (%)	53,757 (58.0%)
Level of education:		
○ Lower	*n* (%)	34,688 (37.6%)
○ Middle	*n* (%)	33,593 (36.4%)
○ Higher	*n* (%)	24,045 (26.0%)
• Partner	*n* (%)	82,020 (88.6%)
*Chronic diseases:*		
• Psychiatric disorders	*n* (%)	19,536 (21.1%)
• Musculoskeletal diseases	*n* (%)	18,401 (19.9%)
• Brain diseases	*n* (%)	18,477 (19.9%)
• Lung disease	*n* (%)	10,652 (11.5%)
• Heart disease	*n* (%)	10,695 (11.5%)
• Endocrine disease	*n* (%)	6177 (6.7%)
• Gastrointestinal diseases	*n* (%)	2106 (2.3%)
• Cancer	*n* (%)	5545 (6.0%)
• Cerebrovascular disease	*n* (%)	1159 (1.3%)
• Kidney disease	*n* (%)	527 (0.6%)
*Lifestyle characteristics:*		
Smoking:		
○ Former smoker	*n* (%)	36,304 (39.5%)
○ Current smoker	*n* (%)	17,186 (18.6%)
• Body mass index (kg/m^2^)	Mean (SD)	26.6 (4.3)
• Alcohol use		
○ No	*n* (%)	21,737 (23.5%)
○ Social	*n* (%)	62,115 (67.0%)
○ Problematic	*n* (%)	8788 (9.5%)
*Frailty:*		
• Frailty index—64 deficits version	mean (SD)	0.13 (0.06)
• Frailty index—35 deficits version	mean (SD)	0.11 (0.06)

The following variables had missing data: level of education (*n* = 314, 0.34%), partner status (*n* = 36, 0.04%), smoking (*n* = 438, 0.47%), and body mass index (*n* = 35, 0.04%)

All analyses were conducted in SPSS version 24. *p* values below .05 were considered statistically significant.

## Results

### Construction of the LifeLines-FI

The baseline characteristics of all participants are shown in Table 1. Of the 92,640 people, a total of 49,076 were 40–49 years of age, 25,023 people were 50–59 years of age, 14,496 people were 60–69 years of age, and lastly, 4045 people were 70 years of age or older.

The FI-64 was slightly positively skewed (skewness 1.17 (SE = 0.01); kurtosis 2.11 (SE = 0.02); median 0.12; range 0.00–0.57). According to the prespecified cutoff values, a total of 17,786 (19.2%) people could be classified as robust, 69,828 (74.5%) people as pre-frail, and 5026 (5.4%) people as frail according to the FI-64. Frailty severity correlated significantly with age in both males and females (Pearson’s *r* = 0.21, *p* < .001). Among people aged 65 and over, these prevalence rates were 6.8% (608/8883), 82.4% (7317/8884), and 10.8% (958/8883), respectively.

**Fig. 1:**
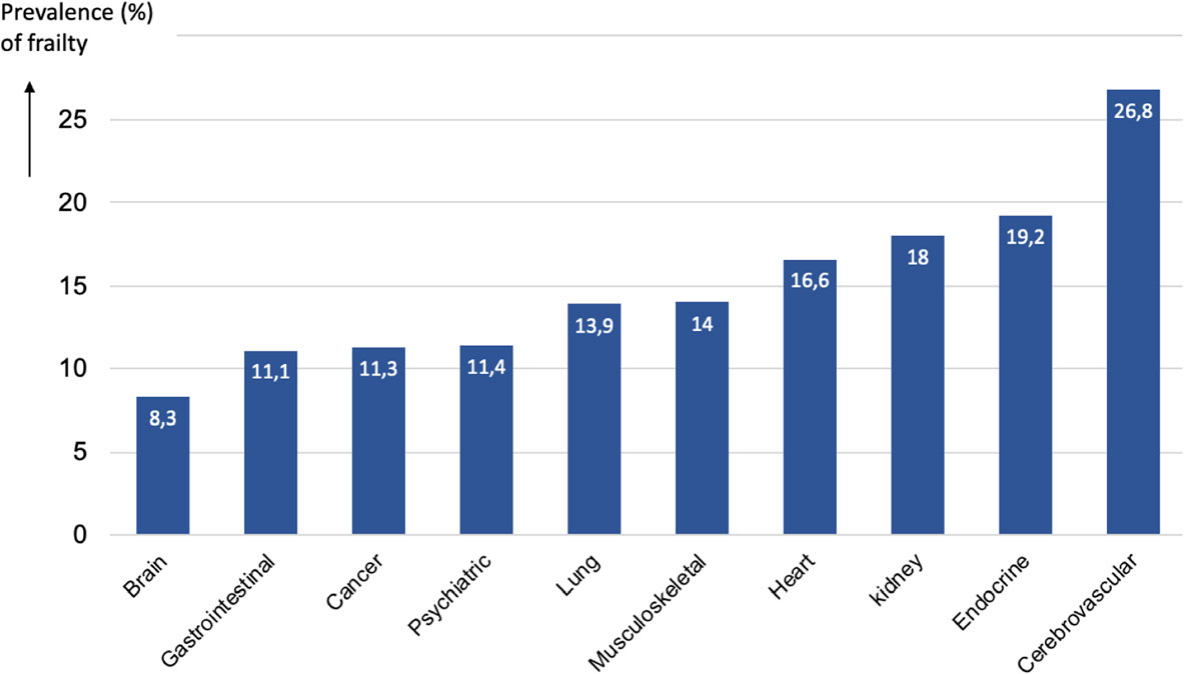
Prevalence of frailty (baseline FI-64 ≥ 0.25) for each chronic disease cluster

Females were significantly more often frail in comparison with males (5.7% versus 5.1%; *χ*^2^=16.1; df = 1, *p* < .001). The prevalence of frailty was lower with an increasing educational level, i.e., 8.7% among low-educated people, 4.2% among medium educated people, and finally 2.5% among people with high education (*χ*^2^=1225.8, df = 2, *p* < .001). Figure 1 presents frailty prevalence rates per chronic disease cluster.

### Association between the FI-64 and 10-year mortality

At the 10-year follow-up, a total of 2092 (2.3%) had died. As shown in Table 2, the FI-64 was associated with mortality independent of lifestyle characteristics and multimorbidity.

**Table 2:** Associations of the FI-64 and chronic disease clusters with 10-year all-cause mortality

	Cox-regression analyses, model 1*			Cox-regression analyses, model 2**		
	HR	[95% CI]	***p***	HR	[95% CI]	***p***
**Separate models per determinant:**						
1. Frailty index *100 (range 1–100)	1.05	[1.05–1.06]	< .001	1.05	[1.05–1.06]	< .001
2. Frailty (yes/no)	2.20	[1.96–2.48]	< .001	2.02	[1.75–2.31]	< .001
3. Multimorbidity (range 0–10)	1.17	[1.12–1.21]	< .001	1.14	[1.10–1.19]	< .001
4. Psychiatric disorders	1.18	[1.06–1.33]	.004	1.10	[0.97–1.24]	.137
5. Musculoskeletal diseases	0.95	[0.86–1.05]	.331	1.01	[0.89–1.12]	.889
6. Brain diseases	0.97	[0.85–1.10]	.598	0.99	[0.87–1.13]	.915
7. Lung disease	1.27	[1.12–1.44]	< .001	1.20	[1.03–1.38]	.016
8. Heart disease	1.24	[1.12–1.38]	< .001	1.16	[1.02–1.32]	.022
9. Endocrine disease	1.24	[1.09–1.42]	.002	1.10	[0.93–1.29]	.283
10. Gastrointestinal diseases	1.17	[0.91–1.51]	.220	1.26	[0.94–1.60]	.126
11. Cancer	1.95	[1.73–2.18]	< .001	2.25	[1.98–2.57]	< .001
12. Cerebrovascular disease	1.65	[1.33–2.04]	< .001	1.65	[1.33–2.04]	< .001
13. Kidney disease	1.91	[1.32–2.78]	.001	1.46	[1.11–1.93]	.008
**Combined model frailty with multimorbidity:**						
• Frailty index *100	1.05	[1.05–1.06]	< .001	1.05	[1.04–1.06]	< .001
• Multimorbidity	0.99	[0.95–1.03]	.568	1.00	[0.95–1.05]	.876
**Combined model frailty with all disease clusters:**						
• Frailty (yes/no)	2.01	[1.76–2.29]	< .001	1.84	[1.56–2.17]	< .001
• Psychiatric disorders	1.08	[0.96–1.21]	.209	0.99	[0.87–1.13]	.897
• Musculoskeletal diseases	0.84	[0.75–0.93]	.001	0.88	[0.78–0.99]	.033
• Brain diseases	0.92	[0.81–1.04]	.198	0.94	[0.82–1.07]	.358
• Lung disease	1.13	[0.99–1.28]	.069	1.09	[0.94–1.26]	.267
• Heart disease	1.08	[0.97–1.21]	.162	1.04	[0.91–1.19]	.549
• Endocrine disease	1.04	[0.91–1.20]	.554	0.99	[0.84–1.18]	.993
• Gastrointestinal diseases	1.10	[0.85–1.41]	.481	1.13	[0.86–1.48]	.375
• Cancer	1.84	[1.64–2.06]	< .001	2.16	[1.89–2.47]	< .001
• Cerebrovascular disease	1.34	[1.07–1.66]	.009	1.25	[0.94–1.66]	.124
• Kidney disease	1.59	[1.10–2.32]	.015	1.79	[1.21–2.65]	.003

*Model 1 is adjusted for age, gender, and level of education

**Model 2 is additionally adjusted for lifestyle characteristics (partner status, smoking, alcohol use, BMI)

The statistically significant association between multimorbidity and mortality disappeared when added simultaneously to the FI-64 in a single model. Pearson’s correlation (*r*) between multimorbidity and the FI-64 was 0.49 (*p* < .001).

Psychiatric disorders, lung disease, heart disease, endocrine disease, cancer, cerebrovascular disease, and kidney diseases were all associated with increased mortality rates (in separate models), which could be explained by lifestyle characteristics in the case of psychiatric disorders and endocrine diseases (see Table 2). When all disease clusters and frailty (yes/no) were added simultaneously into a single model, only cancer and kidney disease were independent predictors of mortality, in addition to frailty.

All of the associations presented in Table 2 were subsequently tested for their interaction with age and gender in separate models for each determinant.

Chronological age interacted only with cancer (*p* < .001) in its association with mortality. Age-stratified results showed that in the fully adjusted models the impact on mortality decreased with increasing age in people with cancer (middle-aged people: HR = 2.87 [95% CI 2.43–3.40], *p* < .001; older people: HR = 1.42 [95% CI 1.22–1.67], *p* < .001).

Gender interacted with the cerebrovascular disease (*p* = .012) only in predicting mortality. Gender-stratified results showed only significant impact in males (males: HR = 1.69 [95% CI 1.31–2.18], *p* < .001; females: HR = 0.98 [95% CI 0.62–1.55], *p* = .979).

Lastly, the FI-64 interacted with lung disease (*p* = .017), heart disease (*p* = .010), and endocrine disease (*p* = .044) in their associations with mortality. Stratified results showed that the impact of frailty was higher in the presence of lung disease (present: HR = 1.07 [95% CI 1.05–1.08], *p* < .001; absent: HR = 1.05 [95% CI 1.04–1.05], *p* < .001), in the presence of heart disease (present: HR = 1.07 [95% CI 1.05–1.08], *p* < .001 versus absent HR = 1.04 [95% CI 1.04–1.05], *p* < .001), and in the presence of endocrine disease (present: HR = 1.06 [95% CI 1.04–1§.08], *p* < .001 versus absent HR = 1.05 [95% CI 1.04–1.05], *p* < .001).

### Course of frailty over a 5-year follow-up

The FI-35 was available for 55,416 people at baseline and 5-year follow-up. People with missing data at 5-year follow-up were significantly more frail at baseline (FI-64: 0.14 (SD = 0.07) versus 0.13 (SD = 0.06), *t* = − 17.3, df = 92,638, *p* < .001), younger (50.8 (SD = 9.1) versus 51.8 (SD = 8.8), *t* = 15.5, df = 96,638, *p* < .001), and less often female (56.8% versus 58.9%, *χ*^2^ = 40.4, df = 1, *p* < .001).

The FI-35 at baseline presented a normal distribution (skewness 0.66 (SE = 0.01); kurtosis 0.81 (SE = 0.02); median 0.11; range 0.00–0.47). The Pearson’s correlation between the FI-64 and the FI-35 was 0.84 (*p* < .001).

Of the 17,244 (31.1%) robust people at baseline, a total of 6256 (36.3%) became pre-frail and 15 (0.1%) became frail at follow-up. Most of the 37,041 pre-frail people at baseline remained pre-frail, but 4351 (11.7%) became robust and 1633 (4.4%) became frail. Also, the majority of the 1131 (2.0%) frail people at baseline remained frail at follow-up, but 452 (40.0%) became pre-frail and none of them became robust.

A paired *t* test showed a significant increase of the FI-35 over time (baseline 0.11 (SD = 0.06); follow-up 0.12 (SD = 0.06), *t* = − 51.7, df = 55,415, *p* < .001). Linear regression with the FI-35 at follow-up as the dependent variable and adjusted for the baseline FI-35 showed that all covariates independently contributed to the change in frailty (baseline FI-35: *ß* = 0.64, *p* < .001; age: *ß* = 0.11, *p* < .001; female gender: *ß* = − 0.05, *p* < .001; compared to lower education: *ß* = − 0.02, *p* < .001 for middle education and *ß* = − 0.04 for higher education; no partner: *ß* = − 0.02, *p* < .001; higher BMI: *ß* = 0.12, *p* < .001; current smoker: *ß* = 0.06, *p* < .001; ex-smoker: *ß* = 0.02, *p* < .001; compared to no alcohol: *ß* = − 0.03, *p* < .001 for moderate alcohol use and *ß* = − 0.02, *p* < .001 for problematic alcohol use).

As shown in Table 3, all chronic disease clusters were significantly associated with an accelerated increase of frailty over the 5-year follow-up. With the exception of gastrointestinal diseases, this effect was independent of comorbidity with other chronic disease clusters (combined model) and also independent of lifestyle characteristics.

**Table 3:** Associations of multimorbidity with progression of frailty over a 5-year follow-up (entire sample age 40+)

	Linear regression, model 1*			Linear regression, model 2**		
Frailty at follow-up predicted by:	***B*** (SE)	Beta	***p*** value	***B*** (SE)	Beta	***p*** value
**Separate models per determinant:**						
1. Multimorbidity (0–10)	0.62 (0.02)	0.10	< .001	0.69 (0.02)	0.11	< .001
2. Psychiatric disorders	0.40 (0.05)	0.03	< .001	0.39 (0.05)	0.02	< .001
3. Musculoskeletal diseases	0.77 (0.05)	0.05	< .001	0.96 (0.05)	0.06	< .001
4. Brain diseases	0.19 (0.05)	0.01	< .001	0.25 (0.05)	0.02	< .001
5. Lung disease	1.23 (0.06)	0.06	< .001	1.23 (0.06)	0.06	< .001
6. Heart disease	0.92 (0.06)	0.05	< .001	0.97 (0.06)	0.05	< .001
7. Endocrine disease	1.21 (0.08)	0.05	< .001	1.09 (0.07)	0.04	< .001
8. Gastrointestinal diseases	0.23 (0.12)	0.01	.052	0.31 (0.12)	0.01	.008
9. Cancer	0.55 (0.08)	0.02	< .001	0.85 (0.08)	0.03	< .001
10. Cerebrovascular disease	1.48 (0.17)	0.03	< .001	1.59 (0.17)	0.03	< .001
11. Kidney disease	1.79 (0.24)	0.02	< .001	2.05 (0.23)	0.02	< .001
**Combined model:**						
• Psychiatric disorders	0.33 (0.05)	0.02	< .001	0.31 (0.04)	0.02	< .001
• Musculoskeletal diseases	0.81 (0.05)	0.05	< .001	1.01 (0.05)	0.06	< .001
• Brain diseases	0.12 (0.05)	0.01	.008	0.18 (0.04)	0.01	< .001
• Lung disease	1.19 (0.06)	0.06	< .001	1.25 (0.06)	0.06	< .001
• Heart disease	0.82 (0.06)	0.04	< .001	0.88 (0.06)	0.04	< .001
• Endocrine disease	1.21 (0.07)	0.05	< .001	1.09 (0.07)	0.04	< .001
• Gastrointestinal diseases	0.13 (0.12)	< 0.01	.284	0.20 (0.12)	0.01	.088
• Cancer	0.67 (0.08)	0.03	< .001	1.01 (0.08)	0.04	< .001
• Cerebrovascular disease	1.44 (0.17)	0.02	< .001	1.57 (0.16)	0.03	< .001
• Kidney disease	1.72 (0.23)	0.02	< .001	2.01 (0.23)	0.02	< .001

*Model 1 is adjusted for baseline frailty severity, age, gender, and level of education

**Model 2 is additionally adjusted for lifestyle characteristics (partner status, smoking, alcohol use, BMI)

Furthermore, multimorbidity, as shown in the separate model in Table 3, had a larger impact on the increase in the FI-35 than any of the diseases separately.

## Discussion

In this large population-based cohort study, we showed that among adults aged ≥ 40 years, frailty is associated with mortality independent of multimorbidity and independent of specific physical or mental disease clusters. In people with either lung or heart diseases, the association of frailty with mortality becomes significantly stronger. While frailty shares “only” 25% variance with multimorbidity in our sample, the association between multimorbidity and mortality was not retained when frailty was added to the model. This shows that the FI explains more variance in mortality than can be explained by a simple disease count and thus is more than merely a measure of multimorbidity [12]. Frailty could be a final common pathway for premature death due to chronic diseases, since all chronic disease clusters were associated with an accelerated increase of frailty over time. This hypothesis implies that prevention of frailty should start at middle age, when the first chronic diseases emerge. These findings add to the first studies of frailty trajectories in very large samples, which show a gradual increase of frailty severity from middle-age onwards [13, 14], with a steeper increase after the age of 65 [19]. Moreover, the FI may gain importance for public health, because its electronic version is being implemented increasingly in routine care (https://www.england.nhs.uk/ourwork/clinical-policy/older-people/frailty/efi/).

The basic findings on the LifeLines-FI are in accordance with previous published evidence. Prevalence rates were significantly higher among females compared to males, correlated with age, and resulted in a prevalence of 10.8% (958/8883) among people aged 65+ years [20]. The Pearson’s correlation between both versions of the LifeLines-FI (FI-64 and the FI-35) was high. This could be expected based on previous findings that the characteristics of a frailty index are independent of the number and type of health deficits included [21].

The prospective association of the FI with mortality was independent of multimorbidity or single-disease clusters but appeared to be significantly stronger in males compared to females. Nevertheless, the clinical importance of this small gender-difference appears to be negligible. Previously, the FI was a better mortality predictor in comparison with chronological age, other frailty instruments such as the Fried’s frailty phenotype model, and other biomarkers of aging [12, 21–23]. The dimensional nature of the FI also has the potential to show subtle changes in frailty status over time [24]. This sensitivity of change appears to be better for tracking risk states over time in comparison with multimorbidity or a simple disease count. Collectively, these findings make the FI an ideal candidate for public health policy in regard to risk prediction and preventive strategies for important health outcomes such as mortality.

Single-disease-based estimates of survival and treatment guidelines are still common, while during the aging process, single-disease clusters may interact with each other and become less accurate for estimating mortality [1]. Most clusters of chronic diseases were associated with an increased 10-year mortality risk, except musculoskeletal, brain, and gastrointestinal diseases. Mortality was independent of frailty only for cancer and kidney disease. Independent effects of frailty and cancer may be explained by the inevitably life-limiting effects of cancer itself. Nevertheless, since frailty increases the likelihood of adverse effects of cancer treatment, and cancer treatment itself may contribute to the emergence of frailty, an independent effect of frailty on mortality in cancer patients is also logical. Several studies have indeed demonstrated the predictive value of the FI for mortality among cancer patients [25]. Whether the increased mortality risk in kidney disease that we found is a chance finding or could be explained by comorbidity with diabetes and/or cardiovascular diseases deserves further study.

Current evidence on the FI in younger adult samples is scarce (e.g., [19, 22, 26, 27]), and in larger samples limited to the UK Biobank study [13, 14]. Data from ~ 500,000 middle-aged and older people who participated in the UK Biobank cohort showed that both an adapted version of the frailty phenotype [13] as well as the FI based on 49 self-report health deficits [14] are associated with mortality. Interestingly, the association of the FI with mortality was even stronger in middle-aged compared to older people in the UK Biobank cohort [13]. The association between the frailty phenotype and mortality was independent of lifestyle and multimorbidity [13], but these data were not reported for the FI [14]. Our study adds that the impact of frailty is largely independent of underlying specific chronic diseases, except for lung and heart disease, which disproportionally increase the mortality risk associated with frailty.

Chronic diseases commonly emerge at younger ages, and their natural course can contribute to the onset and progression of frailty [1]. To our knowledge, this is the first study that demonstrates that all chronic disease clusters, except gastrointestinal diseases, are associated with an accelerated increase of the FI over time, after adjusting for demographic and lifestyle characteristics. The combined model, including all physical and mental disease clusters simultaneously, yielded nearly similar results indicating that the impact of specific chronic diseases on the course of frailty is independent of their initial comorbidity. Moreover, with respect to multimorbidity, we found that the larger the number of different chronic diseases that were present, the steeper the increase of frailty over time. This information is important since patients with single or multiple disease clusters are a heterogeneous group in clinical practice, indicating the idea that a single illness or even multimorbidity could not precisely express prognostication. After the age of 40, even low levels of frailty appear to be more accurate for predicting mortality than the presence of specific chronic diseases.

Some of the strengths of this paper are the large number of middle-aged and older people, as well as a 5-year follow-up tracking of frailty and up to 10-year follow-up in terms of mortality. A strong characteristic of the FI is that its actual performance is independent of the number and types of health deficits included, as long as it has been constructed according to the guidelines described by Searle et al. [11, 21, 28]. This characteristic enables researchers and clinicians to compare results identified by the FI across different cohort studies.” In addition to many previous papers on the FI, we adjusted not only for demographic characteristics, but also for lifestyle characteristics, including partner status, physical activity, alcohol use, smoking, and body mass index. Since lifestyle characteristics hardly affected the strength of the associations in our study, it would be interesting to explore how much variance in frailty trajectories is predetermined by genetic variance.

However, some limitations need to be acknowledged. First, the presence of chronic diseases (and thus multimorbidity) was based on self-report data and we had no information regarding disease severity. Secondly, we did not account for polypharmacy burden, diet habits or biomarkers, which could mediate the association of multimorbidity or single diseases with mortality. Thirdly, regarding the tracking of frailty, we had to rely on an abbreviated 35-item version of the FI instead of the 64-item version that we could only construct at baseline. Attrition was also considerable (54,416/92,640, i.e., 59.8%) at 5-year follow-up. Finally, only 1.5% of the participants were classified as non-Western immigrants, based on the father’s or mother’s country of birth. Therefore, results were neither adjusted for nor stratified by ethnicity, which limits generalizability to ethnic groups other than Caucasians.

## Conclusion

From a public health perspective, we recommend that more studies explore the natural history of frailty among younger adults, including associated negative health outcomes. Many questions remain unanswered to advocate routine assessment of frailty starting among middle-aged people as recommended for older people, especially issues regarding treatment and reversibility of frailty at middle age and cost-effectiveness of case-finding. While multimorbidity may better predict demand for health-related services [29], measurement of frailty in middle-aged and older adults might provide a tool to estimate mortality risk better than single-disease states or multimorbidity. Since frailty is considered a dynamic condition, more knowledge on prevention is also warranted [30].

## Supplementary Information

**Additional file 1.**

## Data Availability

The dataset supporting the conclusions of this article is stored at the LifeLines Biobank server, section OV19-0521, and available upon request (data management LifeLines Biobank, see www.lifelines.nl).
