# Supplementary material for: Course of frailty stratified by physical and mental multimorbidity patterns: a 5-year follow-up of 92,640 participants of the LifeLines cohort study
Source: BMC Med. 2021 Feb 8;19:29. doi: 10.1186/s12916-021-01904-x (PMC7869455; doi:10.1186/s12916-021-01904-x)
Supplement: Supplementary file 1 — Additional file 1. [file 12916_2021_1904_MOESM1_ESM.docx]

**Supplementary material**

**Appendix 1**

Items included in the frailty index (FI-64 and FI-35) of the LifeLines cohort study

| **Health deficits** | **Cut-off values and/or scoring rules** | **FI-64** | **FI-35** |
| --- | --- | --- | --- |
| *Chronic diseases (self-report)* |  |  |  |
| - Cancer | No = 0; yes =1 (defined as having had cancer ever, independent of type) | 1 | 1 |
| - Diabetes type I or II | No = 0; yes = 1 | 2 | 2 |
| - Arthrosis | No = 0; yes = 1 | 3 | 3 |
| - Rheumatoid arthritis | No = 0; yes = 1 | 4 | 4 |
| - Osteoporosis | No = 0; yes = 1 | 5 | 5 |
| - Chronic Obstructive Pulmonary Disease | No = 0; yes = 1 | 6 | 6 |
| - Hypertension | No or don’t know = 0; yes = 1 | 7 | - |
| - Stroke | No = 0; yes = 1 | 8 | 7 |
| - Heart attack | No = 0; yes = 1 | 9 | 8 |
| - Heart failure | No or don’t know = 0; yes = 1 | 10 | 9 |
| - Heart rhythm disorder | No = 0; “have you ever had complaints of an irregular or increased heartbeat?”, and if yes, also confirmation of “Has a doctor diagnosed a heart rhythm disorder?” = 1 | 11 | - |
| - Kidney disease | No = 0; ever had a kidney disease = 1 | 12 | 10 |
| - Morbus Crohn or Colitis Ulcerosa | No = 0; yes = 1 | 13 | - |
| - Incontinence | No = 0; yes = 1 | 14 | - |
| - Number of prescribed drugs (different ATC-codes at the 3-digit level, excluding ATC D). | 0, 1 or 2 = 0; 3 or 4 = 0.5; ≥5 = 1 | 15 | - |
| *Physical performance (physical examination)* |  |  |  |
| - Body mass index (kg/m^2^) | 18.5-25 = 0; 25.1-30 = 0.5;  <18.5 = 1; >30 = 1 | 16 | 11 |
| - Waist circumference (cm) | <88 (men) and <102 (women) = 0; ≥88 (men) and ≥102 (women) = 1 | 17 | 12 |
| - Systolic Blood Pressure (mmHg) | 90-140 = 0; 140-160 = 0.5; <90 = 0.5; >160 = 1 | 18 | 13 |
| - Heart rate (beats/minute) | 60-100 (normal) = 0; <60 (bradycardia) = 0.5 ; >100 (tachycardia) = 1 | 19 | 14 |
| - QTc time on ECG (ms) | ≤450 (men) and ≤470 (women) = 0; >450 (men) and >470 (women) = 1 | 20 | 15 |
| *Activities of daily living (self-report)* |  |  |  |
| - Limitation climbing one stair | Not limited = 0; yes, a little bit limited = 0.50; yes, severely limited = 1 | 21 | - |
| - Limitation lifting/carrying | Not limited = 0; yes, a little bit limited = 0.50; yes, severely limited = 1 | 22 | - |
| - Limitation moderate activities | Not limited = 0; yes, a little bit limited = 0.50; yes, severely limited = 1 | 23 | - |
| - Limitation vigorous activities | Not limited = 0; yes, a little bit limited = 0.50; yes, severely limited = 1 | 24 | - |
| - Limitation walking 100 metre | Not limited = 0; yes, a little bit limited = 0.50; yes, severely limited = 1 | 25 | - |
| - Limitation walking >1 kilometre | Not limited = 0; yes, a little bit limited = 0.50; yes, severely limited = 1 | 26 | - |
| *Blood biomarkers (fasten blood sample)* |  |  |  |
| - Creatinine (umol/L) | ≤110 = 0; >110 = 1 | 27 | 16 |
| - Potassium (mmol/L) | 3.5 - 5.0 = 0; <3.5 = 1; >5.0 = 1 | 28 | 17 |
| - Glucose (mmol/L) | 7.8 - 11.0 = 0; <7.8 = 1; >11.0 =1 | 29 | 18 |
| - Leucocytes (x10^9^/L) | 4.0 - 10.0 = 0; <4.0 = 1; >10.0 = 1 | 30 | 19 |
| - Neutrophils (x10^9^/L) | 1.80 - 7.00 = 0; <1.80 = 1; >7.00 = 1 | 31 | - |
| - Monocytes (x10^9^/L) | 0.30 - 0.90 = 0; <0.30 =1; >0.90 =1 | 32 | 20 |
| - Basophils (x10^9^/L) | ≤0.20 = 0; >0.20 = 1 | 33 | - |
| - Eosinophils (x10^9^/L) | ≤0.40 = 0; >0.40 = 1 | 34 | 21 |
| - Lymphocytes (x10^9^/L) | 0.80 – 3.20 = 0; <0.80 = 1; >3.20 = 1 | 35 | 22 |
| - Erythrocytes (x10^9^/L) | 4.0 – 6.0 = 0; <4.0 = 1; >6.0 = 1 | 36 | 23 |
| - Thrombocytes (x10^9^/L) | 150 - 400 = 0; <150 = 1; >400 = 1 | 37 | 24 |
| - Hb (mmol/L) | 7.5 – 11.0 = 0; <7.5 = 1; >11.0 = 1 | 38 | 25 |
| - HDL cholesterol (mmol/L) | ≥1.55 = 0; <1.55 = 1 | 39 | 26 |
| - LDL cholesterol (mmol/L) | < 2.59 = 0; 2.59 – 3.34 = 0.5 ; >3.34 = 1 | 40 | 27 |
| - Triglycerides (mmol/L) | ≤2.00 = 0; >2.00 = 1 | 41 | 28 |
| *Symptoms (self-report)* |  |  |  |
| - Dizziness/faintness (past week) | No = 0; a little = 0.25; some 0.50; a lot = 0.75; severely = 1 | 42 | 29 |
| - Heavy feelings arms/legs (past week) | No = 0; a little = 0.25; some 0.50; a lot = 0.75; severely = 1 | 43 | 30 |
| - Numbness or tingling in some body parts (past week) | No = 0; a little = 0.25; some 0.50; a lot = 0.75; severely = 1 | 44 | 31 |
| - Chest pain (past week) | No = 0; a little = 0.25; some 0.50; a lot = 0.75; severely = 1 | 45 | 32 |
| - Low back pain (past week) | No = 0; a little = 0.25; some 0.50; a lot = 0.75; severely = 1 | 46 | 33 |
| - Soreness of muscles (past week) | No = 0; a little = 0.25; some 0.50; a lot = 0.75; severely = 1 | 47 | 34 |
| - Difficulty breathing (past week) | No = 0; a little = 0.25; some 0.50; a lot = 0.75; severely = 1 | 48 | 35 |
| - Bodily pain ( “How much pain in the past four weeks?”) | None or very mild = 0; mild = 0.25; moderate 0.50; severe = 0.75; very severe = 1 | 49 | - |
| *Sensory functioning (self-report)* |  |  |  |
| - Are you limited by your eyesight in daily life? | No = 0; yes, a little bit = 0.50; yes, a lot = 1 | 50 | - |
| - Are you limited by your hearing in daily life? | No = 0; yes, a little bit = 0.50; yes, a lot = 1 | 51 | - |
| *Mental functioning (self-report)* |  |  |  |
| - Feeling depressed past two weeks | No = 0; yes = 1 | 52 | - |
| - Loss of interest/anhedonia past two weeks | No = 0; yes = 1 | 53 | - |
| - Feeling happy (past 4 weeks) | None of the time = 1; a little of the time = 0.80; some of the time = 0.60; a good bit of the time = 0.40; most of the time = 0.20; all of the time = 0 | 54 | - |
| - Feeling nervous (past 4 weeks) | None of the time = 0; a little of the time = 0.20; some of the time = 0.40; a good bit of the time = 0.60; most of the time = 0.80; all of the time = 1 | 55 | - |
| - Feeling tired (past 4 weeks) | None of the time = 0; a little of the time = 0.20; some of the time = 0.40; a good bit of the time = 0.60; most of the time = 0.80; all of the time = 1 | 56 | - |
| - Feeling worn out (past 4 weeks) | None of the time = 0; a little of the time = 0.20; some of the time = 0.40; a good bit of the time = 0.60; most of the time = 0.80; all of the time = 1 | 57 | - |
| - Having a lot of energy (past 4 weeks) | None of the time = 1; a little of the time = 0.80; some of the time = 0.60; a good bit of the time = 0.40; most of the time = 0.20; all of the time = 0 | 58 | - |
| *Cognitive functioning (MMSE)* |  |  |  |
| - Orientation in time (MMSE) | Five correct = 0, one wrong = 0.50, two or more wrong = 1 | 59 | - |
| - Orientation in place (MMSE) | Five correct = 0, one wrong = 0.50, two or more wrong = 1 | 60 | - |
| - Attention (MMSE) | Five correct = 0, one or two wrong = 0.50, three or more wrong = 1 | 61 | - |
| - Recall (MMSE) | Three correct = 0, two correct = 0.50, one or zero correct = 1 | 62 | - |
| Subjective functioning |  |  |  |
| - Opinion on your own health | Excellent = 0; very good = 0.25; good = 0.50; fair = 0.75; poor = 1 | 63 | - |
| - I am as healthy as anybody I know | Definitely true = 0; mostly true = 0.25; don’t know = 0.5; mostly false = 0.75; definitively false = 1 | 64 | - |

Abbreviations: MMSE, Mini Mental State Examination; ECG, ElectroCardioGram
